# Is there a justification for classifying GLP-1 receptor agonists as basal and prandial?

**DOI:** 10.1186/s13098-017-0204-6

**Published:** 2017-01-18

**Authors:** Inka Miñambres, Antonio Pérez

**Affiliations:** 1 Endocrinology and Nutrition Department, Hospital de la Santa Creu i Sant Pau, Universitat Autònoma de Barcelona, San Antoni Mª Claret, 167, 08025 Barcelona, Spain; 2 Centro de Investigación Biomédica en Red, Diabetes y Enfermedades Metabólicas Asociadas (CIBERDEM), Madrid, Spain

**Keywords:** Type 2 diabetes mellitus, Glycemic control, Postprandial glycemia, GLP-1 receptor agonists

## Abstract

Several GLP-1 receptor agonists are currently available for treatment of type 2 diabetic patients. Based on their pharmacokinetic/pharmacodynamic profile, these drugs are classified as short-acting GLP-1 receptor agonists (exenatide and lixisenatide) or long-acting GLP-1 receptor agonists (exenatide-LAR, liraglutide, albiglutide, and dulaglutide). In clinical practice, they are also classified as basal or prandial GLP-1 receptor agonists to differentiate between patients who would benefit more from one or another based on characteristics such as previous treatment and the predominance of fasting or postprandial hyperglycemia. In the present article we examine available data on the pharmacokinetic characteristics of the various GLP-1 agonists and compare their effects with respect to the main parameters used to evaluate glycemic control. The article also analyzes whether the differences between the different GLP-1 agonists justify their classification as basal or prandial.

## Background

In contrast to most previous recommendations on the treatment of patients with type 2 diabetes, which were generally aimed at harmonizing and reducing variability in clinical practice, current guidelines advocate an individualized approach [1]. Such an approach affects the choice of medication and the setting of objectives for control of glycemia, taking into account the impact of comorbid conditions, age, the patient’s attitudes and wishes, available resources, and support systems. The patient thus becomes a “partner” in decision-making under the guidance of an experienced health care professional. In the absence of contraindications, metformin continues to be the glucose-lowering drug of choice. Selection of a second agent for combination with metformin requires the physician to weigh up the advantages and disadvantages of each drug for the patient, taking into account individual needs and characteristics.

In the context of personalized treatment as a key strategy in the management of patients with type 2 diabetes, the increasing availability of glucagon-like peptide (GLP) 1 receptor agonists with different pharmacokinetic properties means that the appropriateness of the drug considered should be based on a series of factors: the patient’s specific needs and characteristics, pharmacokinetic properties, antihyperglycemic efficacy, effects on associated processes, and safety profile. A certain degree of controversy surrounds the attempt to classify GLP-1 receptor agonists not only as short- and long-acting, but also as basal and prandial, with the aim of basing the selection on whether the primary objective is to control basal or postprandial hyperglycemia.

The present article examines available data on the pharmacokinetic characteristics of the various GLP-1 agonists and compares their effects with respect to the main parameters used to evaluate glycemic control. The article also aims to consider whether the differences between the different GLP-1 agonists and their application in the treatment of patients with type 2 diabetes justify their classification as basal and prandial.

### Role of basal and prandial hyperglycemia in the treatment of type 2 diabetes

HbA1c is considered to play a key role in the development and progression of the complications of diabetes. Since HbA1c values are determined by both basal and prandial components of hyperglycemia, the most suitable approach to reducing HbA1c in clinical practice should take into account both components [2]. However, the role played by each component varies widely between patients and even within the same patient at different stages of the disease. Furthermore, these roles depend on the degree of glycemic control and the treatment the patient receives [3, 4]. Monnier et al. [3] found that in type 2 diabetic patients managed with diet and/or antidiabetic drugs, the relative contribution of postprandial hyperglycemia was greater in patients with good glycemic control, whereas that of basal hyperglycemia was greater in patients with poor glycemic control. In patients treated with oral antihyperglycemic drugs, Riddle et al. [4] showed that the relative contribution of basal hyperglycemia to HbA1c was 76–80% before intensification with insulin and 31.5–41% after 24–28 weeks of treatment with basal insulin. Moreover, since the main determinant of postprandial hyperglycemia is preprandial hyperglycemia, treatment of basal hyperglycemia is the most efficacious way to control postprandial hyperglycemia [5]. Finally, most major studies on the efficacy of glycemic control are based on fasting blood glucose and HbA1c targets. In addition, a study on patients with type 2 diabetes treated with insulin after acute myocardial infarction showed that insulin therapy focusing on postprandial hyperglycemia provided no benefit over insulin treatment with preprandial glucose targets [6]. These arguments, together with the greater convenience and lower variability and costs of monitoring fasting blood glucose, justify the use of fasting glucose as a means of monitoring treatment. Nevertheless, when basal hyperglycemia is controlled and there is a discrepancy between basal/preprandial values and HbA1c, monitoring and control of postprandial hyperglycemia should be taken into consideration when attempting to achieve HbA1c targets [2].

### Glucose homeostasis: role and differences between GLP-1 receptor analogs

The discovery of the incretin effect arose from the observation that oral administration of glucose generates a more pronounced insulin response than intravenous administration of the same quantity of glucose [7] and accounts for approximately 50–70% of total insulin secretion. The main intestinal peptides responsible for the incretin effect are gastric inhibitory polypeptide (GIP) and GLP-1. Given that the insulinotropic effect of GLP-1 is preserved in individuals with diabetes [8], this hormone has been investigated with the aim of identifying new antihyperglycemic drugs. The currently available drugs that act by boosting the incretin effect comprise dipeptidyl-peptidase-IV (DPP-IV) inhibitors and GLP-1 receptor agonists.

GLP-1 receptor agonists are best known for their insulinotropic effect but only when glucose levels are elevated, thus offering the potential to lower plasma glucose while reducing the risk of hypoglycemia [9]. Furthermore, GLP-1 receptor agonists act against other abnormalities present in individuals with type 2 diabetes and included in the so-called ominous octet of diabetes [10]. Thus, GLP-1 receptor agonists inhibit secretion of glucagon by pancreatic α cells, slow gastric emptying, and induce satiety by acting on the central nervous system [11]. These actions of GLP-1 receptor agonists contribute to their effects on glycemia and weight loss.

In this study, we focus on the differences between the various agonists in terms of pharmacokinetics, pharmacodynamics, and, in particular, on the effects on the parameters used to evaluate glycemic control. Table 1 classifies GLP-1 receptor agonists into short- and long-acting agents and summarizes the main differences between them.

**Table 1:** Main differences between short- and long-acting GLP-1 receptor agonists

	Short-acting	Long-acting
Drugs	Exenatide Lixisenatide	Liraglutide Exenatide-LAR Albiglutide Dulaglutide
Half-life	2–5 h	12 h to several days
Increased secretion of insulin	+	++
Decreased secretion of glucagon	+	+
Decreased gastric emptying	++	+
Decreased fasting plasma glucose	+	++
Decreased postprandial glycemia	+/++^a^	+^a^
Weight loss	+	+/++
Effects (head-to-head studies)		
Reduction in HbA1c		
Exenatide–exenatide-LAR	−1.5% (0.1)	−1.9% (0.1)
Exenatide–liraglutide	−0.79% (0.08)	−1.12% (0.08)
Lixisenatide–liraglutide	Not evaluable^b^	Not evaluable^b^
Exenatide–dulaglutide	−0.99% (0.06)	−1.51% (0.06)
Reduction in fasting plasma glucose		
Exenatide–exenatide-LAR	−1.4 (0.2) mmol/L	−2.3 (0.2) mmol/L
Exenatide–liraglutide	−0.6 (0.2) mmol/L	−1.61 (0.2) mmol/L
Lixisenatide–liraglutide	−0.34 (0.15) mmol/L	−1.3 (0.15) mmol/L
Exenatide–dulaglutide	−1.33 (0.11) mmol/L	−2.39 (0.11) mmol/L
Reduction in hyperglycemia after breakfast		
Exenatide–exenatide-LAR	–6.9 (0.5) mmol/L^c^	–5.3 (0.5) mmol/L^c^
Exenatide–liraglutide	–1.33 mmol/L^d^	
Lixisenatide–liraglutide	–3.9 (0.2) mmol/L^e^	–1.4 (0.2) mmol/L^e^
Exenatide–dulaglutide	Similar reduction	
Reduction in hyperglycemia after lunch and dinner		
Exenatide–exenatide-LAR	Not evaluable	
Exenatide–liraglutide	No differences in meals	
Lixisenatide–liraglutide	Lower glucose levels with liraglutide	
Exenatide–dulaglutide	Higher glucose levels with exenatide	
Weight loss		
Exenatide–exenatide-LAR	–3.6 (0.5) kg	–3.7 (0.5) kg (NS)
Exenatide–liraglutide	–2.87 (0.33) kg	–3.24 (0.33) kg (NS)
Lixisenatide–liraglutide	Not evaluable^b^	Not evaluable^b^
Exenatide–dulaglutide	–1.07 (0.29) kg	–1.3 (0.29) kg

Differences expressed as mean (SD)

Differences are significant unless otherwise indicated (NS)

^a^Postprandial hyperglycemia at meal after administration of the drug

^b^Short duration of the study (28 days)

^c^Change in postprandial glycemia at 2 h

^d^Mean difference between treatments (exenatide–liraglutide)

^e^Change in maximum postprandial glucose excursion

#### Differences in pharmacokinetics/pharmacodynamics

Several GLP-1 receptor agonists are available for treatment of type 2 diabetic patients. The drugs authorized for clinical use include exenatide, exenatide-LAR, liraglutide, lixisenatide, albiglutide, and dulaglutide. Based on their pharmacokinetic/pharmacodynamic profile, these drugs are usually classified as short-acting GLP-1 receptor agonists (exenatide and lixisenatide) or long-acting GLP-1 receptor agonists (exenatide-LAR, liraglutide, albiglutide, and dulaglutide). The main difference between the two groups is that when administered according to their dosing intervals, short-acting agonists are subject to wide fluctuations in the plasma concentration of the active compound, while long-acting agonists exert a more constant effect on the GLP-1 receptor [11].

The natural molecule of human GLP-1 has a plasma half-life of 2–3 min owing to its rapid inactivation by the enzyme DPP-IV [12, 13]. Both exenatide and lixisenatide are synthesized through a modification in the N-terminal portion of the GLP-1 molecule that makes the drugs more resistant to this degradation by DPP-IV and prolongs their half-life to 2–4 h [14]. Although the half-life of both molecules is similar, exenatide is administered every 12 h whereas lixisenatide is taken once daily. In the case of long-acting GLP-1 receptor agonists, the mechanism that enables more stable and prolonged concentrations of the active compound is different. For liraglutide, the addition of a fatty acid chain bound non-covalently to the GLP-1 analog enables the drug to bind to plasma albumin, ensuring that only a small percentage circulates freely and can be eliminated by the kidneys. Thus, the elimination kinetics of liraglutide depends on the rate of dissociation between GLP-1 and albumin, conferring a half-life of approximately 12 h with a single daily subcutaneous dose [15, 16]. Exenatide-LAR, on the other hand, is designed in such a way that the active GLP-1 is encapsulated in poly(d,l lactic-co-glycolic acid) microspheres enabling sustained release of the drug from the subcutaneous reservoir generated at the injection site. Consequently, when the drug is administered weekly, stable concentrations are reached 6–8 weeks after initiation of therapy [17, 18]. Albiglutide is the product of fusion of two modified human GLP-1 [7–36] molecules to albumin. A single amino acid substitution of alanine to glycine at position 8 confers resistance to hydrolysis by DDP-IV and ensures a half-life of approximately 5 days [19]. Finally, dulaglutide consists of two identical chains of N-terminal GLP-1 joined by a disulfide bridge, each of which is bound covalently by means of a small binding peptide to a modified human immunoglobulin heavy chain. This modification confers resistance to degradation by DPP-IV and ensures the sustained release that accounts for the 5-day half-life [20].

The various pharmacokinetic characteristics of the different GLP-1 receptor agonists lead to differences in pharmacodynamics. Long-acting analogs can maintain high GLP-1 levels and stimulate secretion of insulin for 24 h, even during fasting periods, resulting in a greater reduction in basal hyperglycemia. The effect of these GLP-1 receptor agonists on postprandial hyperglycemia can be attributed mainly to suppression of glucagon secretion, reduced appetite, and slower gastric emptying.

The effect of short-acting GLP-1 receptor agonists on insulin secretion during the fasting period is less pronounced than that of long-acting agonists, whereas the effect on gastric emptying is more pronounced [21, 22]. This difference in the effects on gastric emptying between short and long-acting GLP-1 receptor agonists can be attributed to tachyphylaxis, which occurs when stimulation of the receptor is constant—as is the case with long-acting agonists—but not when stimulation is intermittent [23]. The more pronounced effect on gastric emptying is the main reason for the greater efficacy of short-acting agonists in reducing postprandial hyperglycemia in the meal immediately following their administration [24, 25].

#### Effects of GLP-1 agonists on HbA1c and basal and postprandial hyperglycemia

The effects of the various GLP-1 receptor agonists on glucose levels have been analyzed in phase III placebo-controlled randomized trials within the framework of drug development programs such as AMIGO (exenatide), DURATION (exenatide-LAR), Get Goal (lixisenatide), LEAD (liraglutide), HARMONY (albiglutide), and AWARD (dulaglutide). All of these agents reduce HbA1c, basal hyperglycemia, and postprandial hyperglycemia. However, it is difficult to draw comparisons between them, since the studies were performed in different populations. In addition, the variables analyzed and the way they are expressed also differ between the studies. For example, the effects on postprandial hyperglycemia were evaluated after a test meal or after the patient’s usual meals and not always at the same time of the day in all the trials. In some cases, postprandial hyperglycemia is expressed as the peak glucose value after meals, whereas in others it is expressed as postprandial glucose excursion; in addition, the results are expressed as the absolute effect or as the difference with respect to placebo. Consequently, it is extremely difficult to compare findings between studies, and the data provided by recently published reviews [26–29] do not clarify these findings, since the confusion over terminology and the way the results are expressed remains unresolved or is simply disregarded.

Taken together, the different GLP-1 receptor agonists decrease HbA1c by around 1% (0.3–1.3%), whereas the reduction in fasting plasma glucose ranges from 0.2 to 2.1 mmol/L (3.6–37.8 mg/dL). In studies evaluating the effect on postprandial hyperglycemia, the decrease observed was 1.8–6.2 mmol/L (32.4–111.6 mg/dL) [28, 30–32]. The reduction in HbA1c and basal hyperglycemia is generally greater with long-acting agonists whereas the reduction in postprandial hyperglycemia in the meal immediately following administration is greater with short-acting agonists.

In this context, available data from head-to-head studies may help to clarify the differences between the different GLP-1 receptor agonists. In addition, the postprandial effect should be evaluated throughout the day and not only after the first meal of the day, as is the case in some of the trials published.

### Comparison between GLP-1 analogs in head-to-head studies

#### Exenatide vs exenatide-LAR

The DURATION-1 study [17] and DURATION-5 study [33] compared exenatide 10 µg/12 h with exenatide-LAR 2 mg/week. The results of DURATION-1 showed that after 30 weeks of follow-up, the reduction in HbA1c was greater with exenatide-LAR (−1.9 vs −1.5%; p = 0.0023), as was the decrease in fasting plasma glucose (−2.3 vs −1.4 mmol/L; −41.4 vs 25.2 mg/dL; p < 0.05); however, the decrease in postprandial glycemia after a standard breakfast was greater with exenatide than with exenatide-LAR (−6.9 vs −5.3 mmol/L; 124.2 vs 95.4 mg/dL; p = 0.0124). The analysis of gastric emptying evaluated using paracetamol absorption showed that emptying was slower with twice-daily exenatide [17]. The results of DURATION-5 showed that the reduction in HbA1c was greater with exenatide-LAR than with twice-daily exenatide. In this study, the effect on postprandial hyperglycemia was not analyzed [33]. No differences in terms of weight decrease were found in either study.

#### Exenatide vs lixisenatide

GetGoal X was a noninferiority trial comparing lixisenatide with exenatide. The reduction in HbA1c was 0.79 and 0.96%, respectively, and the reduction in fasting plasma glucose was 1.3 and 1.49 mmol/L (23.4 and 26.82 mg/dL), thus confirming that lixisenatide was not inferior to exenatide. Differences in postprandial glycemia were not analyzed, and a greater decrease in body weight was observed with exenatide (−3.98 vs −2.96 kg) [34].

#### Lixisenatide vs liraglutide

In a randomized trial, Kapitza et al. [35] compared the effect of lixisenatide and liraglutide on postprandial hyperglycemia at 28 days. The results show a greater reduction in maximum postprandial hyperglycemia after a standardized breakfast with lixisenatide (−3.9 vs −1.4 mmol/L; −70.2 vs −25.2 mg/dL; p < 0.0001). However, the 24-h pharmacodynamic profile of both drugs at days 1 and 28 showed that despite differences in glucose levels during the first 4 h after breakfast in favor of lixisenatide, from 4.5 h onward, postprandial hyperglycemia was lower in patients treated with liraglutide. Liraglutide led to greater decreases in basal plasma glucose (−1.3 vs −0.3 mmol/L; 23.4 vs 5.4 mg/dL; p < 0.001), HbA1c (−0.51 vs −0.32%; p < 0.01), and body weight (−2.4 vs −1.6 kg; p < 0.001).

#### Liraglutide vs exenatide

The LEAD-6 study compared liraglutide with exenatide in terms of the effects of each on blood glucose parameters [22]. The results showed that liraglutide led to a greater decrease in HbA1c (−1.12 vs −0.79%; p < 0.0001) and fasting plasma glucose (−1.61 vs 0.6 mmol/L; −28.9 vs −10.8 mg/dL; p < 0.0001). Self-monitoring of plasma glucose at 26 weeks showed that the reduction in hyperglycemia after breakfast and dinner was greater with exenatide, with the difference between treatments estimated at 1.33 mmol/L (23.9 mg/dL) after breakfast (p < 0.0001) and 1.01 mmol/L (18.2 mg/dL) after dinner (p < 0.0005). No differences were observed in glucose levels after lunch or in weight loss.

#### Liraglutide vs exenatide-LAR

The DURATION-6 study showed that liraglutide was more efficacious than exenatide-LAR in reducing HbA1c (−1.48 vs −1.28%; p = 0.0018) and basal plasma glucose (−2.12 vs −1.76 mmol/L; −38.16 vs −31.68 mg/dL; p < 0.001). Liraglutide also led to greater weight loss than exenatide-LAR (−3.57 kg vs −2.68 kg; p < 0.001). The effect on postprandial hyperglycemia was not studied [36].

#### Albiglutide vs liraglutide

In the HARMONY 7 study [37], patients who received once-daily liraglutide had greater reductions in HbA1c (−0.99% vs −0.78), fasting plasma glucose (−1.68 vs −1.22 mmol/L; −30.24 vs 21.96 mg/dL; p < 0.05), and weight loss (−2.19 vs −0.64 kg; p < 0.0001) than did those who received once-weekly albiglutide. No data were provided for postprandial hyperglycemia.

#### Dulaglutide vs exenatide

In the AWARD-1 study [30], dulaglutide led to a greater reduction in HbA1c (−1.51 vs −0.99%; p < 0.001) and fasting plasma glucose (−2.38 vs –1.33 mmol/L; −43 vs −24 mg/dL; p < 0.001). Data from capillary blood glucose monitoring at 26 weeks showed that the reduction in postprandial glycemia at breakfast was similar for both exenatide and dulaglutide, whereas the reduction in postprandial hyperglycemia during the rest of the day was superior with dulaglutide (data not shown). No differences in weight were recorded.

#### Dulaglutide vs liraglutide

The AWARD-6 study [38] showed that the decrease in HbA1c, fasting plasma glucose, and postprandial glycemia was similar with dulaglutide and liraglutide, although weight loss was greater with liraglutide (−2.9 kg vs −3.61 kg; p < 0.05).

### Effect of GLP-1 analogs on daily postprandial hyperglycemia

In order to evaluate the action of a drug on postprandial hyperglycemia, especially when it is not administered before each meal, it is necessary to determine the effect on postprandial hyperglycemia throughout the day, not only immediately after the drug is administered.

In most studies on GLP-1 receptor agonists, the effects on postprandial glycemia are limited to breakfast. However, although available data are scarce, findings from some studies can better explain the effect of the drug at other points during the day. In their randomized study, Lorenz et al. [25] compared the effect of morning lixisenatide with placebo on postprandial hyperglycemia at breakfast, lunch, and dinner. The results showed a significant reduction in postprandial hyperglycemia throughout the day with lixisenatide, although the reduction was very marked at the glucose peak after breakfast (−3.9 ± 0.6 mmol/L; −70.2 ± 10.8 mg/dL) and less marked after lunch (−1.2 ± 0.7 mmol/L; −21.6 ± 12.6 mg/dL) and dinner (−0.6 ± 0.7 mmol/L; −10.8 ± 12.6 mg/dL). Kapitza et al. [35] found lixisenatide to be clearly superior to liraglutide for reducing postprandial glycemia after breakfast [−3.9 vs 1.4 mmol/L (−70.2 vs 25.2 mg/dL) for the change in maximum glucose excursion; p < 0.0001], although capillary glucose monitoring values during the rest of the day were lower with liraglutide, including the values recorded after lunch and dinner. In the LEAD-6 study, which compared liraglutide with exenatide, the reduction in hyperglycemia after breakfast and dinner was greater with exenatide, whereas no differences were observed after lunch [22]. Finally, in the AWARD-1 study [30], the reduction in hyperglycemia after breakfast was similar with exenatide and dulaglutide, whereas the effect of dulaglutide was superior after lunch and dinner. These findings highlight the more pronounced postprandial effect of short-acting analogs, although this more pronounced effect seems to be limited to the meal that is eaten immediately after administration of the drug.

As stated above, after initiation of treatment with basal insulin, which decreases fasting glucose, the relative contribution of postprandial hyperglycemia to total hyperglycemia increases from 20–24% to 59–69% [4]. GLP-1 receptor agonists stimulate glucose-medicated insulin secretion, suppress glucagon secretion, delay gastric emptying, and decrease appetite, thus explaining the considerable effect of these drugs on postprandial hyperglycemia and weight loss. Therefore, patients treated with basal insulin also seem to be suitable candidates for GLP-1 receptor agonists, and combining GLP-1 receptor agonists with basal insulin offers an alternative approach to intensification of insulin therapy when basal insulin is insufficient. Systematic reviews and meta-analyses of prospective studies [39–42] demonstrate that GLP-1 receptor agonists added to basal insulin decrease postprandial glucose levels, HbA1c levels, body weight, and basal insulin requirements without increasing the risk of major hypoglycemic events. Randomized studies that evaluate the effect of GLP-1 receptor agonists combined with basal insulin [43–46] confirm these findings. Buse et al. [43] found that the addition of exenatide to basal insulin led to a reduction in HbA1c that was significantly greater than with placebo (−1.74 vs −1.04%; p < 0.001), with a lower increase in the dose of insulin. In addition, data on self-monitoring of capillary glucose show significant decreases in postprandial glucose levels at breakfast and dinner. In the three Get Goal studies [44–46], addition of lixisenatide to basal insulin led to an improvement in HbA1c and hyperglycemia after breakfast, as well as weight loss. In addition, some randomized controlled trials have compared GLP-1 receptor agonists to prandial insulin in patients treated with a basal insulin regimen. Diamant et al. [47] analyzed the addition of exenatide in patients treated with insulin glargine and found a similar reduction in HbA1c with respect to addition of insulin lispro; however, weight loss and the reduction in fasting plasma glucose were greater with exenatide. Mathieu et al. [48] compared the effect of liraglutide with that of single-dose insulin aspart administered with the main meal of the day in patients who had previously received insulin degludec and metformin. The results revealed a greater reduction in HbA1c and weight loss in patients treated with liraglutide. Finally, the HARMONY-6 study [49], which compared albiglutide with insulin lispro in patients treated with insulin glargine, revealed a similar reduction in HbA1c, but with fewer cases of hypoglycemia and greater weight loss. Overall, as stated in the meta-analysis of Eng et al. [42], compared with basal bolus insulin regimens, the combination of basal insulin and a GLP-1 receptor agonist leads to a mean reduction in HbA1c of −0.1% (−0.17 to −0.02%), with a lower relative risk of hypoglycemia (0.67; 0.56 to 0.80) and a reduction in mean weight loss (−5.66 kg; −9.8 to −1.51).

The data presented here show the efficacy of short-term treatment with GLP-1 receptor agonists compared with placebo or short-acting insulin in patients taking basal insulin. However, our findings are limited by the lack of data on the long-term effects of GLP-1 receptor agonists used in combination with insulin. Furthermore, data enabling us to predict which individuals will respond or not to GLP-1 agonists are very limited [50].

There is emerging data suggesting efficacy differences between GLP-1 receptor agonists according to pancreatic insulin reserve. In a recent post hoc study of GetGoal-M and getGoal-S trials, lixisenatide has demonstrated its efficacy across different stages of beta cell dysfunction [51], while long-term efficacy of liraglutide seems to be related to β-cell function [52]. These findings may reflect the fact that short-acting analogs exert most of their effects on glycemia through the decrease in gastric emptying as stated before [24, 25], whereas long-acting analog effects are more dependent of insulin secretion.

Therefore, the possibility of differentiating between early responders and nonresponders to different GLP-1 receptor agonists and establishing the efficacy and safety of these agents in the long term has yet to be clarified. In addition, their role in patients receiving basal bolus insulin regimens warrants further study.

### Effects of GLP-1 receptor agonists on cardiovascular outcomes

At this time, there are three published CV safety outcome trials for the GLP-1 receptor agonists: lixisenatide in acute coronary syndrome (ELIXA) trial [53], liraglutide effect and action in diabetes: evaluation of CV outcome results (LEADER) [54], and trial to evaluate CV and other long-term outcomes with semaglutide in subjects with type 2 diabetes (SUSTAIN-6) [55] conducted with lixisenatide, liraglutide, and semaglutide and with a median follow-up of 2.1, 3.8 and 2.0 years, respectively. All of these studies were powered to assess non-inferiority or to adequately detect differences between the drug and placebo on the 3-point major adverse cardiac events (MACE; cardiovascular death, nonfatal myocardial infarction, or nonfatal stroke). The ELIXA trial included 6068 patients with type 2 diabetes who had experienced an acute coronary event within 180 days prior to randomization, and LEADER and SUSTAIN-6 trials enrolled 9340 and 3297 adults with type 2 diabetes at high cardiovascular risk, respectively. The ELIXA study demonstrated cardiovascular safety, but not cardiovascular benefit of the short acting GLP-1 RA lixisenatide, while LEADER and SUSTAIN-6 trials showed that the long-acting GLP-1 receptor agonists liraglutide and semaglutide reduce cardiovascular risk.

The mechanisms for improved cardiovascular outcomes with liraglutide and semaglutide but not with lixisenatide are not clear. All studies showed improved glycemic control, reduced body weight and systolic blood pressure, and increased heart rate. Moreover, differences in baseline patient characteristics, trial duration, and routine care do not seem to account for differences in cardiovascular outcomes. Previous studies showed possible beneficial effects on endothelial function and the CV system through direct effects mediated through GLP-1 receptor-dependent and through independent mechanisms [56]. Unlike those treated with liraglutide and semaglutide, patients treated with short-acting GLP-1 RA lixisenatide are uncovered by the drug for most of the day. Thus, pharmacokinetic differences could play a role in the lower incidence of adverse CV outcomes with liraglutide and semaglutide, and its confirmation in the currently underway trials with other long-acting GLP-1 RA, would support a class effect of long-acting GLP-1 RA for cardiovascular outcomes.

## Conclusions

This study raised the question of whether the differences between the different GLP-1 receptor agonists and their application in the treatment of patients with type 2 diabetes support their classification as basal and prandial. Given the available pharmacological and clinical data, we believe the most appropriate classification of GLP-1 receptor agonists is as short-term and long-term agents. Both pharmacokinetic and pharmacodynamic data support this classification and make it possible to differentiate between some of the drugs’ clinical effects. However, given the considerable differences between available agents and those currently under development, in the future it would probably be more appropriate to classify GLP-1 receptor agonists as short-acting, intermediate-acting, and long-acting.

In contrast, unlike basal and prandial insulin, whose effects are clearly different, currently available data make it somewhat difficult to support the classification of GLP-1 receptor agonists into basal and prandial. In fact, if we were to draw an analogy, GLP-1 receptor agonists would be closer to intermediate-acting insulins, since both have a basal and prandial component that can vary over a 24-h period. To a greater or lesser extent, all GLP-1 receptor agonists affect both the basal and prandial components of hyperglycemia in patients with type 2 diabetes, although the main mechanism by which they reduce postprandial hyperglycemia can differ between drugs and with the same drug administered at different mealtimes during the day. Therefore, the reduction in postprandial glycemia with short-acting agonists (exenatide, lixisenatide) seems to be more associated with gastric emptying during the meal immediately after administration. With long-acting agonists, however, most of the decrease in postprandial glycemia probably results from the decrease in preprandial glycemia—the main component of postprandial hyperglycemia—and the glucagon suppression that is common to all GLP-1 receptor agonists. The effect on preprandial hyperglycemia may also be the most relevant mechanism of short-acting receptor agonists in the reduction of postprandial hyperglycemia at meals where the drug is not administered.

In addition, as mentioned above, in most patients with type 2 diabetes who are candidates for a second or third antihyperglycemic drug, the main abnormality is basal hyperglycemia [4]. In this context, control of basal glycemia is the most efficacious way to treat postprandial glycemia [5]. Nevertheless, in patients treated with basal insulin and in cases where the glycemic profile shows a clear predominance of prandial hyperglycemia, a drug with a more specific action against this abnormality could prove useful.

In conclusion, GLP-1 receptor agonists cannot replace basal insulin or prandial insulin. Data enabling us to identify responders and nonresponders are scarce and, although the effect on glucagon suppression, gastric emptying, and appetite probably persists in patients with marked insulin deficiency, we do not have data on the durability of efficacy of GLP-1 receptor agonists during the course of type 2 diabetes. Therefore, continuation or discontinuation of treatment should be decided on an individual basis depending on the clinical response.

## Abbreviations

GLP-1: glucagon-like peptide 1
GIP: gastric inhibitory polypeptide
DPP-IV: dipeptidyl-peptidase-4
AMIGO: AC2993 diabetes management for improving glucose outcomes
LEAD: liraglutide effect and action in diabetes
DURATION: diabetes therapy utilization: researching changes in A1C, weight and other factors through intervention with exenatide once weekly
GetGoal: GLP-1 agonist AVE0010 in patients with type 2 diabetes mellitus for glycemic control and safety evaluation
AWARD: assessment of weekly administration of dulaglutide in diabetes
ELIXA: evaluation of lixisenatide in acute coronary syndrome
LEADER: liraglutide effect and action in diabetes evaluation of cardiovascular outcome results
SUSTAIN-6: trial to evaluate cardiovascular and other long-term outcomes with semaglutide in subjects with type 2 diabetes
EXSCEL: exenatide study of cardiovascular event lowering
REWIND: researching cardiovascular events with a weekly incretin in diabetes
